# Synthesis, Characterization and Mechanical Properties of Novel Bio-Based Polyurethane Foams Using Cellulose-Derived Polyol for Chain Extension and Cellulose Citrate as a Thickener Additive

**DOI:** 10.3390/polym13162802

**Published:** 2021-08-20

**Authors:** Loredana Maiuolo, Fabrizio Olivito, Vincenzo Algieri, Paola Costanzo, Antonio Jiritano, Matteo Antonio Tallarida, Antonio Tursi, Corradino Sposato, Andrea Feo, Antonio De Nino

**Affiliations:** 1 Department of Chemistry and Chemical Technologies, University of Calabria, 87036 Rende, CS, Italy; vincenzo.algieri@unical.it (V.A.); paola.costanzo@unical.it (P.C.); antonio.jiritano@unical.it (A.J.); matteoa.tallarida@unical.it (M.A.T.); antonio.tursi@unical.it (A.T.); 2 ENEA, Italian National Agency for New Technologies, Energy and Sustainable Economic Development, Trisaia Research Centre, S.S. 106 Ionica, km 419 + 500, 75026 Rotondella, MT, Italy; corradino.sposato@enea.it (C.S.); andrea.feo@enea.it (A.F.)

**Keywords:** bio-based polyurethanes, prepolymers, cellulose-derived polyol, cellulose-citrate, polyurethane composites

## Abstract

A novel series of bio-based polyurethane composite foams was prepared, employing a cellulose-derived polyol for chain extension and cellulose-citrate as a thickener additive. The utilized polyol was obtained from the reduction reaction of cellulose-derived bio-oil through the use of sodium borohydride and iodine. Primarily, we produced both rigid and flexible polyurethane foams through chain extension of the prepolymers. Secondly, we investigated the role of cellulose citrate as a polyurethane additive to improve the mechanical properties of the realized composite materials. The products were characterized by FT-IR spectroscopy and their morphologies were analysed by SEM. Mechanical tests were evaluated to open new perspectives towards different applications.

## 1. Introduction

During the last decade, industrial and academic interest in the conversion of renewable biomass into fuels, chemicals and useful materials grew enormously [1,2]. In contrast to the limited fossil sources, agricultural wastes represent an opportunity because they are widely available, with their annually produced quantity estimated at around 2 × 10^11^ tons per year [3,4]. In terms of the future prospect of a totally sustainable economy, chemistry plays a central role [5,6,7]. Cellulose is the major component of natural feedstocks and the most important efforts of the scientific community are focused on the development of new methodologies for its transformation [8,9,10,11]. Pyrolysis is one of the oldest chemical methods to convert cellulose into useful platform molecules such as sugars, furans or bio-oil mixtures [12,13,14]. The harsh conditions related to high temperature and pressure were substituted, over the years, by milder approaches [15,16,17,18]. However, under milder pressure and temperature, hydrolysis is usually accompanied by the use of such mineral acids as sulfuric or hydrochloric acid, and this involves damage to process equipment as well as negative environmental impact [19,20]. Unconventional solvents such as ionic liquids or deep eutectic solvents solved many environmental and procedural problems [21,22], but they are still limited by the difficulty of recovering the products, due to their high affinity for these phases [23,24,25,26]. In this ambit, novel surfactant-based ionic liquids are receiving attention due to their ease of preparation, low cost, and inherent added-value properties; in fact, their (tuneable) amphiphilic nature can, in principle, help in solving affinity problems, but applications are still far from being achieved [27,28]. Ball milling and ultrasounds are the most used physical methods for the pre-treatment and the conversion of cellulosic biomass, but the scalability of these methods is usually limited [29,30]. Cellulose-derived products are extremely versatile building blocks for the production of materials for engineering applications, medical equipment, food-related purposes, biofuels and many other applications [31,32,33]. Polyurethanes (PUs) are multifunctional polymers that have been known and produced for a long time, with wide commercial diffusion due to their low cost and significant versatility, having been used, for example, as thermal insulation materials [34,35]. Conventional PUs can be formed by a very exothermic reaction of isocyanates and polyols [36] but, in recent years, industrial and research efforts have been focused on the production of bio-based products with more eco-sustainable methodologies [37,38,39,40].

Cellulose can be turned into valuable polyols that can be used as a starting materials or chain extenders for polyurethane synthesis [41,42,43]. We recently developed a simple and fast method for the contemporary conversion of microcrystalline cellulose into a furan-enriched bio-oil and cellulose citrate [44]. In another recent work, we proved the efficacy of some inorganic salts, such as sodium chloride, in the reaction of PEG 400 and different aliphatic diisocyanates for the production of polyurethanes [45].

The principal goal of this work was the development of a novel, efficient and sustainable approach for the synthesis of polyurethane foams and polyurethane composites using two renewable materials and a green catalyst. More specifically, the furan-enriched bio-oil obtained through our previous procedure [44] was successfully reduced using a simple open flask reaction into a polyol mixture, composed mainly of 2,5-bis(hydroxyl methyl)furan. More precisely, this furan diol was proven, over the years, to have different applications, and the synthetic efforts towards its facile production are growing rapidly [46,47,48]. For the aforementioned reasons, we used—for the first time, to our knowledge—this cellulose-derived polyol, which was synthesized through our simple and eco-compatible procedure, as a chain extender to synthesize novel bio-based polyurethane foams. Subsequently, the addition of the second renewable product into the same reaction mixture, with cellulose citrate as an additive, allowed us to realize novel polyurethane composite foams with enhanced mechanical properties. The final materials were chemically characterized by FT-IR spectroscopy and their morphologies were analysed by SEM. Their mechanical properties were preliminary evaluated to facilitate new application-based studies using these materials. Finally, a conceptualization regarding the chemical interactions of cellulose citrate inside the urethane chains was carried out.

## 2. Materials and Methods

Tetrahydrofuran (THF) was purchased from Carlo Erba (Milan, Italy) at analytical grade, and freshly distilled before use after drying over sodium sulfate. Acetone was purchased from Honeywell at a high purity grade and used without further purification. Microsrystalline cellulose type 102 was purchased from Roquette (Lestrem, France) at high purity. Citric acid was purchased from Sigma Aldrich (St. Louis, MO, USA) at 99% purity grade. Sodium borohydride was purchased from Carlo Erba (Milan, Italy) at 95% purity grade. Iodine was purchased from Carlo Erba (Milan, Itay) at the analytical grade. Polyethylene glycol (PEG) 400 was purchased from Thermo Fisher Scientific (Waltham, MA, USA) at 99% purity grade. Isophorone diisocyanate (IPDI) **3**, 4,4′-methylenedicyclohexyl diisocyanate (H_12_MDI) **4**, and 2,2,4-trimethyl hexamethylene diisocyanate (TMDI) **5** were purchased from EVONIK INDUSTRIES (Essen, Germany), at 95% purity grade. Sodium chloride and sodium sulfate anhydrous was purchased from Sigma Aldrich (St. Louis, MO, USA) at analytical grade. IR spectra of all products are reported in the Supplementary Material (Sections S2, S3 and S5).

### 2.1. Bio-Oil Reduction into Polyol ***1***

A quantity of 50 g of bio-oil was dissolved in 200 mL of THF dry in a 500 mL one-neck round bottom flask. In addition, 12.5 g (25% in weight respect to bio-oil) of NaBH_4_ was added portion-wise and hydrogen was released from the reaction flask. After one hour, 15 g (30% in weight respect to bio-oil) of iodine was added slowly while the hydrogen was released from the reaction flask. After one hour, the excess NaBH_4_ was quenched with aqueous HCl 1M. The mixture was filtered through a sintered glass funnel. The solution was dried over sodium sulphate, filtered, and the solvent was removed under vacuum to obtain a polyol in the form of a brown oil, with a yield of 85–90%. The LC-MS and FT-IR spectra of bio-oil and polyol are reported in the Supplementary Material (Sections S4 and S5).

### 2.2. Hydroxyl Group Content of Biomass Derived Polyol ***1***

Polyol **1** was initially esterified using phthalic anhydride according to an earlier study [49]. In the same reaction flask, phthalic anhydride (112 g, 0.76 mols) and imidazole (17 g, 0.25 mols) were dissolved in pyridine (700 mL) (this mixture is called phthalation reagent). One gram of polyol and 25 mL of the phthalation reagent were mixed under stirring at 100 °C for 15 min. The mixture was cooled down to room temperature and 50 mL of pyridine and 10 mL of distilled water were added. The tritant solution NaOH (0.5 M) was added until the pink ending point (1 mL of phenol-phthalein solution, 1% *w*/*v* in EtOH, was used as indicator). The experiments were replicated three times and the value expressed as a mean value. The hydroxyl group content was determined using the following equation:

(1) $$Hydroxyl\;number=\frac{\left(B-S\right)\left(56.1\right)\left(N\right)}{W}$$

*B* = the volume of the blank (mL) at the ending point; *S* = the volume of the substrate (mL) at the ending point; *N* = the normality of the NaOH solution; *W* = the weight of the substrate. The OH content of cellulose-derived polyol **1** was 310 mg KOH/g.

### 2.3. Prepolymer Synthesis ***6***–***8***

The detailed procedure is reported in the literature [45]. Briefly, three different prepolymers **6**–**8** were synthesized using the relative diisocyanates **3**–**5** at a molar excess of 2.5:1 with respect to PEG 400 **2**. Water was used as a blowing agent at a weight percentage of 5.6% with respect to PEG 400 **2**. The reagents were added in the following order in a plastic container: PEG 400 **2**, distilled water and NaCl as catalyst. The mixture was stirred using a mechanical apparatus. The appropriate diisocyanate was added and the mixture was vigorously stirred. The blend was warmed up to 70 °C for one hour until diisocyanate consumption. The three prepolymers **6**–**8** were obtained using the same procedure in the form of a colourless gel. The reaction was monitored by FT-IR spectroscopy with respect to the isocyanate signal. The produced prepolymers were defined as stable in accordance with the standard titration method, ASTM D 2572-97, using di-*n*-butylamine [49].

### 2.4. Synthesis of PU Foams ***9***–***11*** Using Polyol ***1*** as a Chain Extender

Polyol **1** at 30% weight with respect to the prepolymer was added to the freshly prepared prepolymer, and the mixture was vigorously and mechanically stirred for a few minutes in the same plastic container. After that, the same mixture was reversed in a steel mold (Supplementary Material, Section S6) and the system was closed under pressure at room temperature. After eight hours, the polyurethane foam was obtained in the final form for further use (see Supplementary Material, Section S7). The disappearance of the isocyanate signal was monitored by FT-IR spectroscopy.

### 2.5. Preparation of PU Composite Foams ***12***–***14*** Using Cellulose Citrate as a Thickener Additive

Polyol **1** at 30% weight and cellulose citrate at 20% weight, with respect to the prepolymer, were added sequentially in the same plastic container as the prepolymer, and the mixture was vigorously and mechanically stirred for a few minutes. After that, the same mixture was reversed in a steel mold and the system was closed under pressure at room temperature. After eight hours, the polyurethane foam was obtained in the final form for further use. The disappearance of the isocyanate signal was monitored by FT-IR spectroscopy.

### 2.6. Fourier-Transform Infrared Spectroscopy (FT-IR)

FT-IR spectra were acquired using the Shimadzu IRAffinity-1S spectrometer (Shimadzu Corporation, Kyoto, Japan) in the spectral region of 400 to 4000 cm^−1^ with a resolution of 1 cm^−1^, with 48 scans undertaken for a single analysis, and the KBr pellets technique used. The KBr pellets were obtained by mixing the substrate with KBr powder (ratio 1:100) and by pressing with a hydraulic press. All spectra are collected in the Supplementary Material (Sections S2, S3 and S5).

### 2.7. Scanning Electron Microscopy (SEM)

Morphological studies of PU composites were carried out using a LEO 420 scanning electron microscope (SEM, Zeiss, Oberkochen, Germany), operating with vacuum conditions of 9 × 10^6^ Torr at an accelerating voltage of 15 kV. Before analysis, all substrates were gold metallized using an Auto Sputter Coater (Agar, Stansted, UK). Images were taken with 100 SEM micrograph magnications. The cell structure analysis was conducted using the Aphelion^TM^ software version 4.5.0. The program, through the use of SEM images, provided the cell surface and cell number. Furthermore, the anisotropy index was calculated as the ratio of cell height to width in the perpendicular direction of the foam growth.

### 2.8. Mechanical Test

Mechanical tests were performed in accordance with UNI EN ISO 3386-2:2010, “Flexible cellular polymeric materials, Determination of stress–strain characteristic in compression. Part 2: High density material”. The test pieces were cubes with square load-bearing surfaces of 40 mm minimum size.

An Instron 3369 double column universal machine with a load cell of 50 kN, equipped with two circular plates of 160 mm in diameter, was used for the test. The compression plate moved at a uniform low rate of 2 mm/min.

## 3. Results and Discussion

**Scheme 1:**
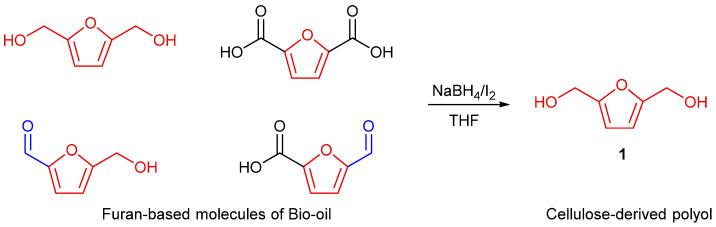
Bio-oil reduction to cellulose-derived polyol.

The conversion of microcrystalline cellulose (MCC) into multi-functional molecules is intricate because of its high ordered crystalline structure and the low accessibility, which limit the interactions with chemicals and the consequent depolymerisation [50,51]. In our previous work, we proved the role of molten citric acid in the contemporary hydrolysis and esterification of MCC [44]. With the aim of producing a renewable polyol, the obtained furan-enriched bio-oil was reduced by a procedure employed for the conversion of carboxylic acids, which makes use of sodium borohydride and iodine, as mild reducing agents, in an open-air system [52]. This reaction is useful for bio-oil reduction because aldehydes and ketones can be reduced by sodium borohydride while the sodium borohydride/iodine system is necessary for the reduction of carboxylic groups. The reaction is shown in Scheme 1.

The newly synthesized polyol **1** was obtained as a dark highly viscous oil following two hours of the aforementioned one-pot reaction, and 2,5-bis(hydroxymethyl)furan was identified as the main compound through the LC-MS technique (see Supplementary Material for FT-IR and LC-MS spectra of polyo **l**, Sections S3 and S4).

It is a common practice for the production of polyurethane foams to use a two-step methodology through which the prepolymers produced in the first step can be extended with short diols in the second step [53]. Initially, we followed our previously reported method for the synthesis of prepolymers using polyethylene glycol (PEG 400) (**2**) with three different aliphatic diisocyanates—isophorone diisocyanate (IPDI) (**3**), 4,4′-methylenedicyclohexyl diisocyanate (H_12_MDI) (**4**) and 2,2,4-trimethyl hexamethylene diisocyanate (TMDI) (**5**)—and sodium chloride as the catalyst [45]. We chose these diisocyanates as source of polyurethanes not only because of their easy commercial availability, but also due to their aliphatic nature. In fact, the aromatic diisocyanates are certainly more commonly used, but they are very toxic. Moreover, few works in the literature that involve the use of aliphatic diisocyanates are reported [54,55], and this fact prompted us to develop an adequate and more eco-friendly synthetic strategy.

**Scheme 2:**
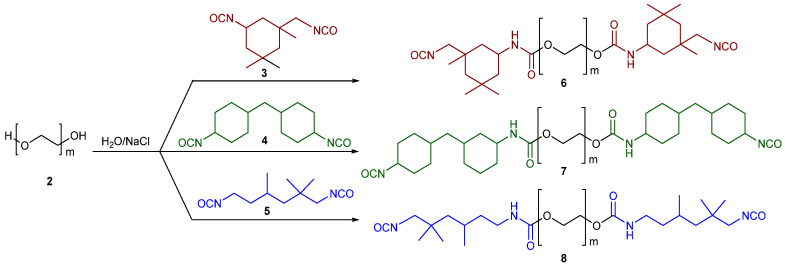
Reaction scheme for the synthesis of the prepolymers (**6–8**).

The reaction products (**6**–**8**) are shown in Scheme 2.

**Scheme 3:**
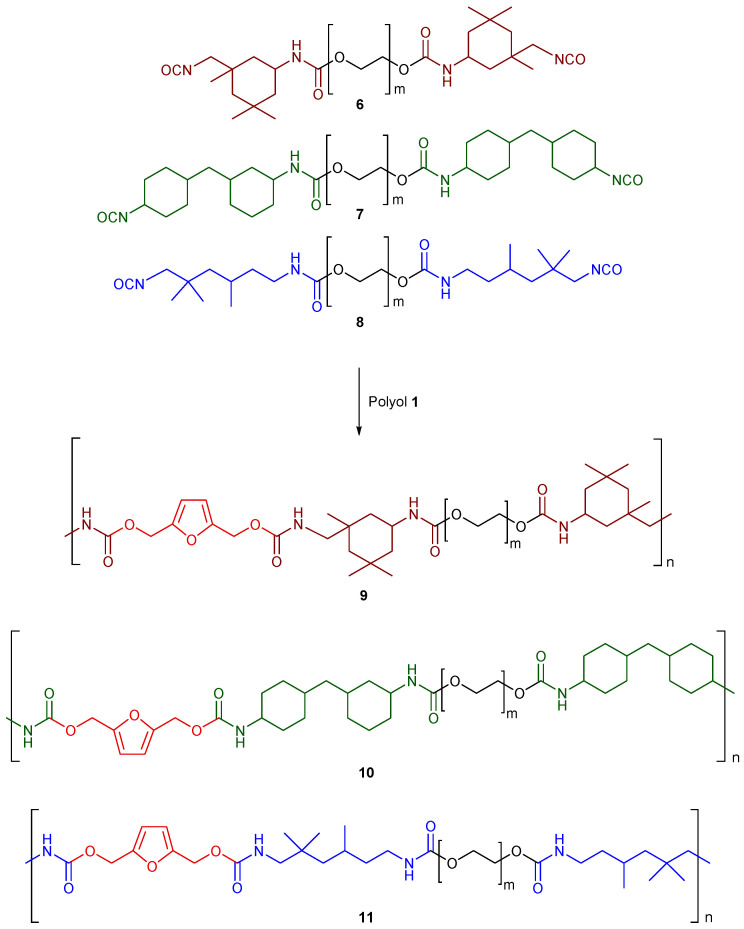
The final products after chain extension with polyol **1**.

Successively, we employed the cellulose-derived polyol (**1**) as a chain extender for the three prepolymers (**6**–**8**) (Scheme 3). The reaction was conducted, without the addition of any ulterior catalyst, in a closed steel mold, at room temperature, and the products (**9**–**11**) were obtained in the form of polyurethane foams after 8 h. The quantitative conversion was verified by FT-IR analysis through the complete disappearance of the isocyanate signal (see Supplementary Material, Section S5).

**Figure 1:**
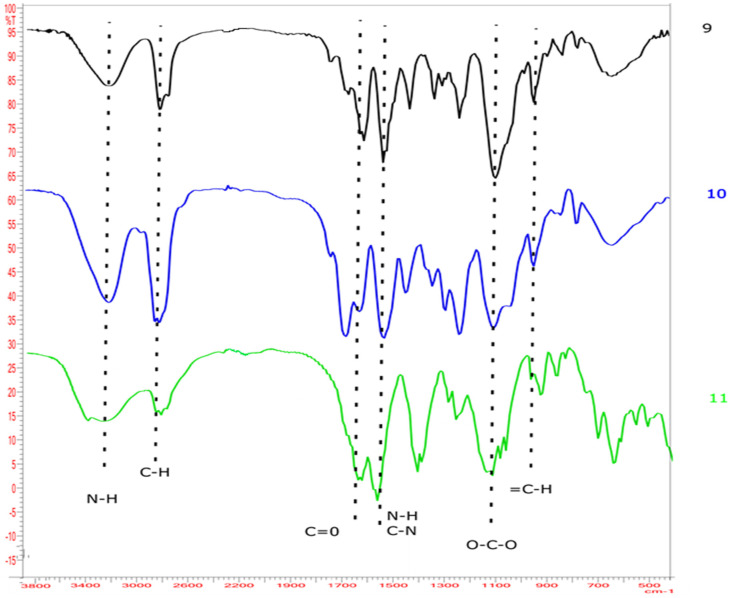
FT-IR spectra of the products (**9–11**).

The properties of the realized composite materials were chemically characterized by FT-IR spectroscopy (Figure 1). We marked the characteristic peaks of the polyurethane structure. The vibrational bands near 3330 cm^−1^ are relative to N-H stretching vibrations. The shoulder at its high-frequency side (around 3600 cm^−1^) can be due to tightly bounded or monomeric residual molecules of water [56]. The IR signal in the region near 3000 cm^−1^ corresponds to both the asymmetrical and symmetrical stretching vibrations of aliphatic C-H. The signal near 1700 cm^−1^ corresponds to C=O stretching vibration. The band above 1500 cm^−1^ is probably relative to N-H bending vibrations and C-N stretching vibrations. The IR signal near 1100 cm^−1^ is relative to O-C-O stretching frequency. The band in the region near 950 cm^−1^ is relative to the =C-H bending vibrations of furan [57,58].

Finally, cellulose citrate was employed in the same polyurethane formulations as an additive and the relative polyurethane composites were obtained (**12**–**14**) (Table 1, Section S8).

**Figure 2:**
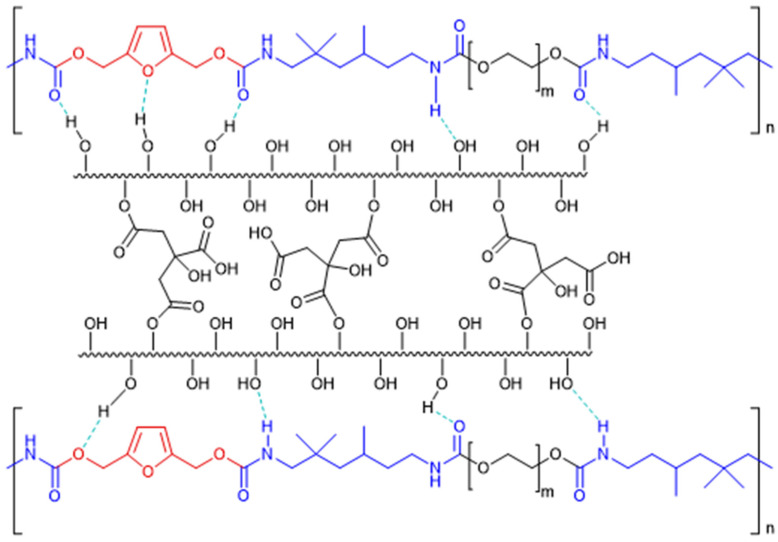
Graphical representation of the composite material **14** derived from cellulose citrate and polyurethane **11**.

For example, Figure 2 shows the interaction forces between cellulose citrate and the chains of polyurethane **14**. Considering the chemical nature of polyurethane chains and cellulose citrate, we suppose a strong interaction between them, assisted by a hydrogen bond network.

**Figure 3:**
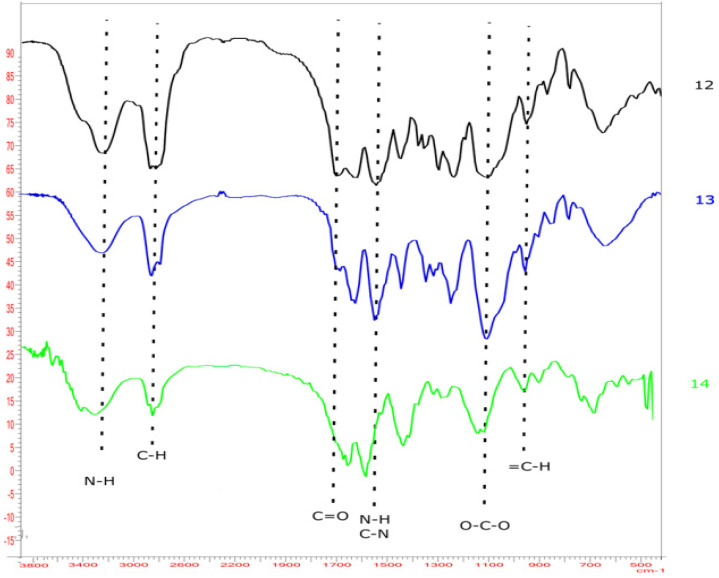
FT-IR spectrum of **14** that can confirm the presence of hydrogen bonds.

The presence of the hydrogen bonds could be confirmed by FT-IR spectra, as can be observed in the spectrum of materials **12**–**14**. In fact, the band in the range of 3600–2800 cm^−1^ is very broad and can also encompass the vibration signal of the -OH group (Figure 3).

Moreover, in the literature, a shift to lower frequencies was documented in the carbonyl group when this group took part in hydrogen bonds [59]. The shift was evident for the C=O stretching of the citrate group, which, from its typical vibration signal near 1735 cm^−1^ in cellulose citrate, was evidently shifted to lower frequency of 1700 cm^−1^ in all of the composites due to the fact that this molecule was engaged in a network of hydrogen bonds.

**Figure 4:**
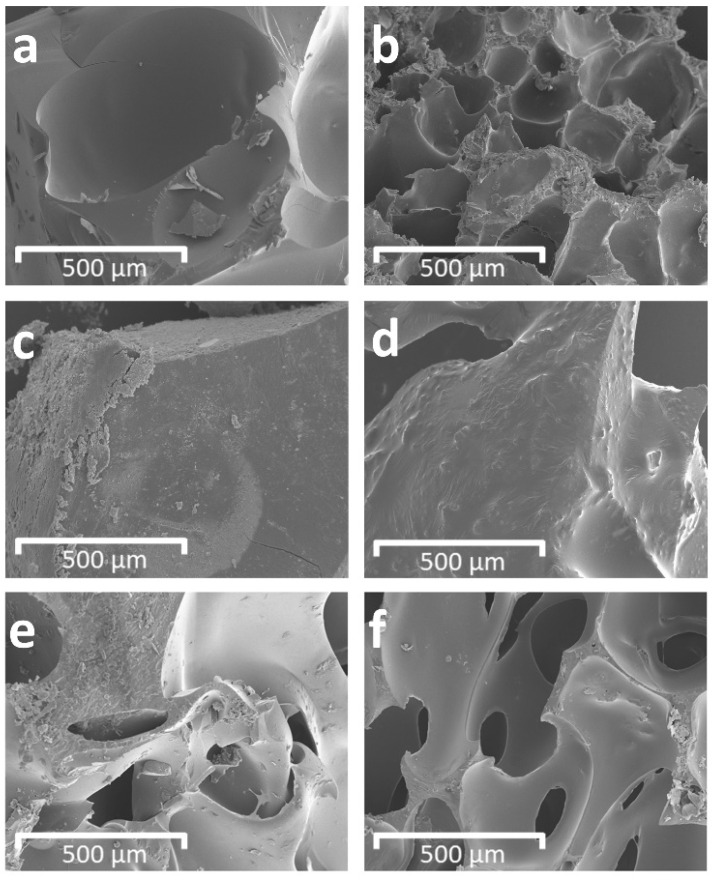
Scanning electron microscopy (SEM) images of structure of PUs and PU composites (×100 magnification): (**a**) **9**, (**b**) **12**, (**c**) **10**, (**d**) **13**, (**e**) **11**, (**f**) **14**.

Scanning electron microscopy observations were carried out to monitor the structure of PUs **9**–**11** and PU composites **12**–**14** (Figure 4). In all cases, the morphological analysis shows the typical polyurethane structure. In the case of **9** (Figure 4a) and **12** (Figure 4b), two types of pores can be observed, large and small, with dimensions of 500 and 150 µm, respectively. Both types of pores are evenly distributed in the matrix. For **10** (Figure 4c) and **13** (Figure 4d), the structure appears compact and devoid of pores; in particular, for **13** (Figure 4d), a roughness, attributable to the presence of cellulose citrate, is observed on its surface. For **11** (Figure 4e) and **14** (Figure 4f), both still have a pore-rich structure with sizes between 250 and 50 µm, but **14** appears more compact than **11 [60]**. Ultimately, the morphological analysis indicates that the presence of cellulose citrate generates a decrease in the size of the pores and/or a greater compactness, with a consequent increase in density, as also found in the mechanical characterizations. These findings could be attributable to the interaction forces between the cellulose citrate and the polyurethane chains. Apart from their mutual chemical affinity, this hypothesis could be reinforced by the results of some studies which pointed out that, in cases of blending of differently sized molecules, a spontaneous structuring takes place, driven by the fact that smaller molecules tend to be more likely to occupy the voids made by bigger ones, with a compression of volume and, overall, a more compact structure [61,62].

Structural parameters were determined, and are included in Table 2.

The anisotropy index is defined as the ratio of the length to the width of the cell. When this ratio is close to 1, the shape of the cell approaches a sphere [63]. The cells of **9**, **11**, **12** and **14** in the cross section, which were perpendicular to the direction of the foam growth, were characterized by anisotropy index very close to 1. These values indicate that the cells have almost spherical shapes. Therefore, the introduction of cellulose citrate into the systems does not change the shape of the cells, considering that the values of the anisotropy index are similar in all systems. It was observed that, due to the nucleation effect, the addition of cellulose citrate for PU composites **12** and **14** increased the number of cells in the cross section of the foam, as compared to the corresponding additive-free PU systems **9** and **11**, respectively. The effect of increasing the number of cells after adding cellulose citrate or other fillers was reported in the literature [64,65]. For **10** and **13**, the evaluation of the characteristics of the cellular structures was not performed as they appeared compact and devoid of pores, as can be seen in Figure 4c,d.

Finally, a comparison of the mechanical properties of the PUs **9**–**11** and the PU composites **12**–**14** was carried out [37,60]. The results are reported in Table 3.

Products **10** and **13** show a high density, of about 1000 kg/m^3^, and a brittle behaviour. The material, during the compression test, fractured without significant plastic deformation. The maximum values of the stress–strain curves are 0.95 MPa and 1.1 MPa for **10** and **13**, respectively. The compounds **9** and **12** show lower density (about 290 kg/m^3^), and better compressive strength (compared to compounds **10** and **13**), with a leathery behaviour. In the stress–strain curve, after an initial linear loading regime, the foam specimens show relatively abrupt yielding, followed by a sustained plateau region. Finally, **11** and **14** have a rubbery behaviour and a density comparable to **9** and **12**. For this type of material, the breaking load has not been evaluated, as they are elastomers; in fact, they deform during the compression test and, after the load is released, they return to their initial dimensions.

## 4. Conclusions

In conclusion, bio-based composite materials were successfully synthesized and characterized. The syntheses were carried out by two-step routes by using, in the second step (elongation stage), a furan-enriched polyol, derived from cellulose, as a chain extender to synthesize eco-sustainable polyurethane foams. In particular, our efforts made it possible to reduce bio-oil in an innovative manner, in an open-air reaction flask and under atmospheric pressure, to obtain a furan-enriched polyol, a privileged renewable platform molecule with a wide range of applications in the present and for the future. We used this product and we proved that is an optimal chain extender for this kind of polymer. For the second series of composites, cellulose citrate, another renewable material, was employed as a thickener for the first time, and it was added into the same reaction mixture to obtain novel composite materials with enhanced mechanical properties. In fact, from preliminary mechanical data, it seems that the use of cellulose citrate as an additive improves the mechanical performance of the materials, increasing their compressive strength. Moreover, the experimental data confirm that the hyperbranched structure of the cellulose citrate introduced into polyurethane chains increases the hydrogen bonds in the PU system (see Figure 2), causing a compression of volume and a more compact structure in the composite materials. In general, prepared bio-based polyurethane foams and their composites show good mechanical properties; therefore, we believe that our innovative method should be considered very attractive. Certainly, ulterior studies will be necessary in order to establish other properties of our materials—such as thermal conductivity and thermal stability—for such applications as insulators.

In the future, we can consider the potential use of our products in the industrial sector, and in agrifood and agroforestry waste processing, as a source of cellulose citrate and furan-enriched polyol, with strong environmental impact due to the recycling of waste material. Therefore, a wider application of this simple, efficient and eco-sustainable methodology may represent a valid alternative to other elaborate processes that are currently in use; moreover, the production of novel bio-based polyurethane foams and polyurethane composites, with their chemical and physical properties, will surely be informative for future applications in this research field.

## Figures and Tables

**Table 1:** Polyurethane composite materials **12–14**.

PU	Chain Extender	Additive	PU Composite
**9**	Polyol **1**	Cellulose citrate	**12**
**10**	Polyol **1**	Cellulose citrate	**13**
**11**	Polyol **1**	Cellulose citrate	**14**

**Table 2:** Characteristics of PU cellular structure.

PU	Anisotropy Index	Number of Cell/(mm^2^)	Average Cell Area (mm^2^)
**9**	0.97 ± 0.05	21 ± 2	0.242 ± 0.044
**11**	1.09 ± 0.12	8 ± 2	0.503 ± 0.063
**12**	1.12 ± 0.06	109 ± 7	0.023 ± 0.008
**14**	1.16 ± 0.07	39 ± 4	0.152 ± 0.012

**Table 3:** Mechanical properties of PUs **9–11** and PU composites **12–14**.

Substrates	Density (kg/m^3^)	Compressive Strength (MPa)
**9**	260 ± 10	2.03 ± 0.04
**10**	970 ± 10	0.95 ± 0.05
**11**	270 ± 10	-
**12**	290 ± 10	2.41 ± 0.09
**13**	1000 ± 10	1.10 ± 0.06
**14**	280 ± 10	-

## Data Availability

Data are contained within the article.

## Supplementary Materials

The following are available online at https://www.mdpi.com/article/10.3390/polym13162802/s1. Section S1: Reagents; Section S2: FT-IR spectra of PEG 400, isocyanates and the relative prepolymers; Section S3: FT-IR spectra of biomass derived polyol (1); Section S4: LC-MS spectrum of polyol (1); Section S5: FT-IR spectra of biomass-based polyurethanes; Section S6: Composite material processing equipment; Section S7: Photographs of bio-based polyurethanes; Section S8: Photographs of bio-based composite materials.
