# Supplementary material for: Synthesis, Characterization and Mechanical Properties of Novel Bio-Based Polyurethane Foams Using Cellulose-Derived Polyol for Chain Extension and Cellulose Citrate as a Thickener Additive
Source: Polymers (Basel). 2021 Aug 20;13(16):2802. doi: 10.3390/polym13162802 (PMC8400649; doi:10.3390/polym13162802)
Supplement: Supplementary file 1 [file polymers-13-02802-s001.zip › polymers-1330689-supplementary.pdf]

Supplementary Material

Synthesis, Characterization and Mechanical Properties of Novel Bio-based Polyurethane Foams Using Cellulose-derived Polyol for Chain Extension and Cellulose Citrate as a Thickener Additive

Loredana Maiuolo [1,*], Fabrizio Olivito [1,*], Vincenzo Algieri [1], Paola Costanzo [1], Antonio Jiritano [1], Matteo Antonio Tallarida [1], Antonio Tursi [1], Corradino Sposato [2], Andrea Feo [2] and Antonio De Nino [1,*]

[1] Department of Chemistry and Chemical Technologies, University of Calabria, 87036 Rende, CS, Italy; vincenzo.algieri@unical.it (V.A.); paola.costanzo@unical.it (P.C.); antonio.jiritano@unical.it (A.J.); matteoa.tallarida@unical.it (M.A.T.); antonio.tursi@unical.it (A.T.)

[2] ENEA, Italian National Agency for New Technologies, Energy and Sustainable Economic Development, Trisaia Research Centre, S.S. 106 Ionica, km 419 + 500, 75026 Rotondella, MT, Italy; corradino.sposato@enea.it (C.S.); andrea.feo@enea.it (A.F.)

* Correspondence: maiuolo@unical.it (L.M.); fabrizio.olivito@unical.it (F.O.); denino@unical.it (A.D.N.)

Table of Contents

Section S1: Reagents **S3**

Section S2: FT-IR spectra of PEG 400, isocyanates and the relative prepolymers **S4**

Section S3: FT-IR spectra of biomass-derived polyol (1) **S11**

Section S4: LC-MS spectrum of polyol (1) **S12**

Section S5: FT-IR spectra of biomass-based polyurethanes **S13**

Section S6: Composite material processing equipment **S19**

Section S7: Photographs of bio-based polyurethanes **S20**

Section S8: Photographs of bio-based composite materials **S21**

Section S1. Reagents

Polyethylene glycol PEG 400 (Thermo Fisher Scientific)
Molecular formula: $H(OCH_2CH_2)_nOH$, n = 8-9 (average)
Appearance (Color): Clear colorless
Form: Viscous liquid
Identification (FTIR): Conforms
Molecular Weight: Average mol weight 380-420 (based on OH value)
Hydroxyl Value: 267- 295 mg KOH/g
pH: 4.5-7.0 (10%, demin water)
Viscosity: 104-140 mPa·s at 20°C

Isophorone diisocyanate IPDI (EVONIK INDUSTRIES, Code: VESTANAT^{R}IPDI)
Molecular formula: $C_{12}H_{18}N_2O_2$
Appearance (Color): Clear colorless
Form: liquid
Identification (FTIR): Conforms
Molecular Weight: 222.3 g/mol
NCO content: 37.5- 37.8 %
Viscosity: 14 mPa·s at 20°C
Density: 1.058 g/ml

4,4′-dicyclohexylmethane diisocyanate H_{12}MDI (EVONIK INDUSTRIES, Code: VESTANAT^RH_{12}MDI)
Molecular formula: $C_{15}H_{22}N_2O_2$
Appearance (Color): Clear colorless
Form: liquid
Identification (FTIR): Conforms
Molecular Weight: 262.35 g/mol
NCO content: 31.8- 32 %
Viscosity: 35 mPa·s at 20°C
Density: 1.07 g/ml

Trimethylhexamethylene diisocyanate TMDI (EVONIK INDUSTRIES, Code: VESTANAT^{R}TMDI)
Molecular formula: $C_{11}H_{18}N_2O_2$
Appearance (Color): Clear colorless
Form: liquid
Identification (FTIR): Conforms
Molecular Weight: 210.27 g/mol
NCO content: 39.7- 40 %
Viscosity: 5-8 mPa·s at 20°C
Density: 1.01 g/ml

Sodium Chloride NaCl (Sigma Aldrich, 99%)

Section S2. FT-IR spectra of PEG 400, isocyanates and the relative prepolymers

FT-IR spectrum of PEG 400 (2)

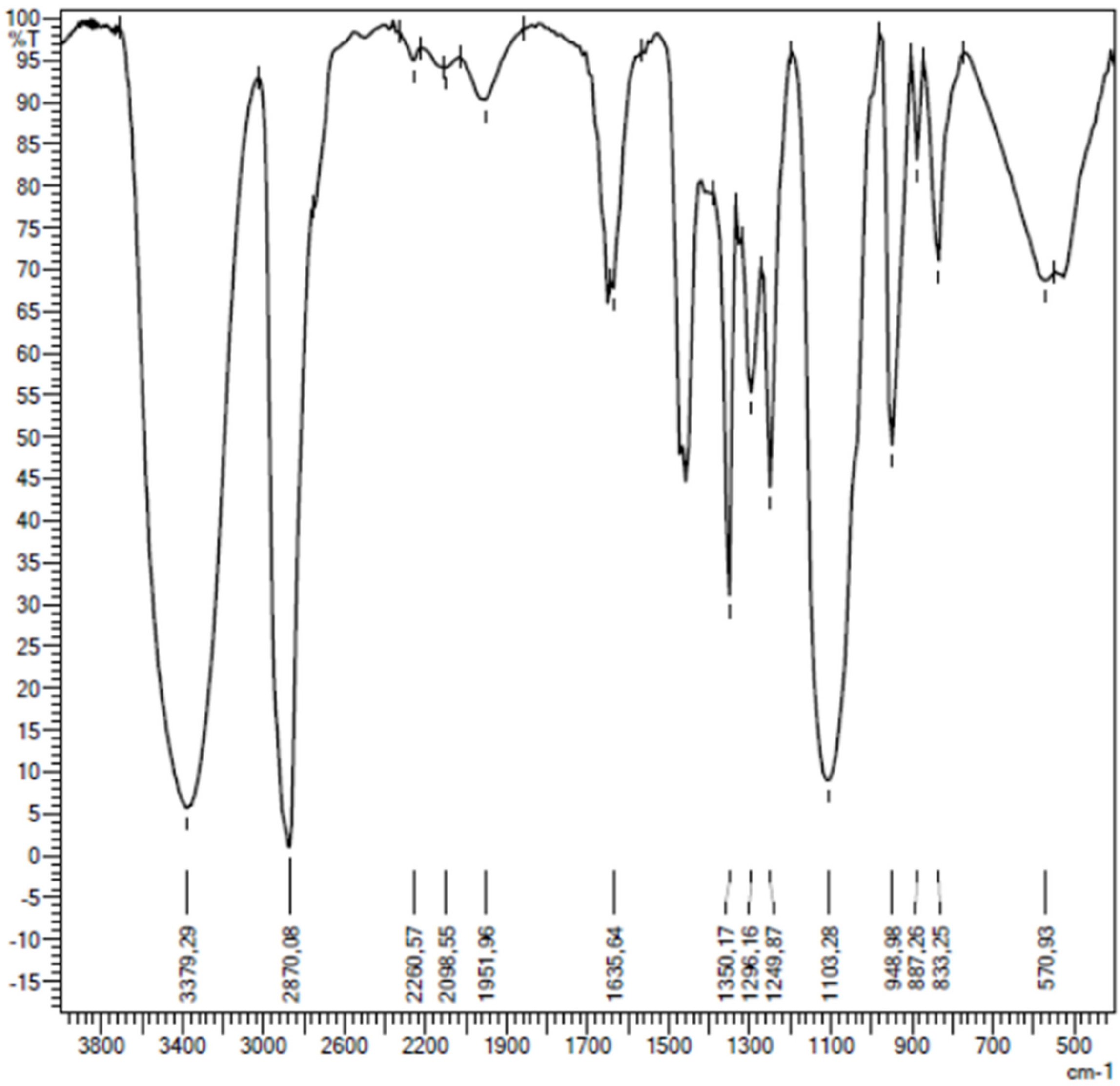

Group (stretching)	Absorption peak (cm^{-1})
O-H	3379
C-H	2870
C-O-C and C-O-H	1050-1180

FT-IR spectrum of isophorone diisocyanate (IPDI) (3)

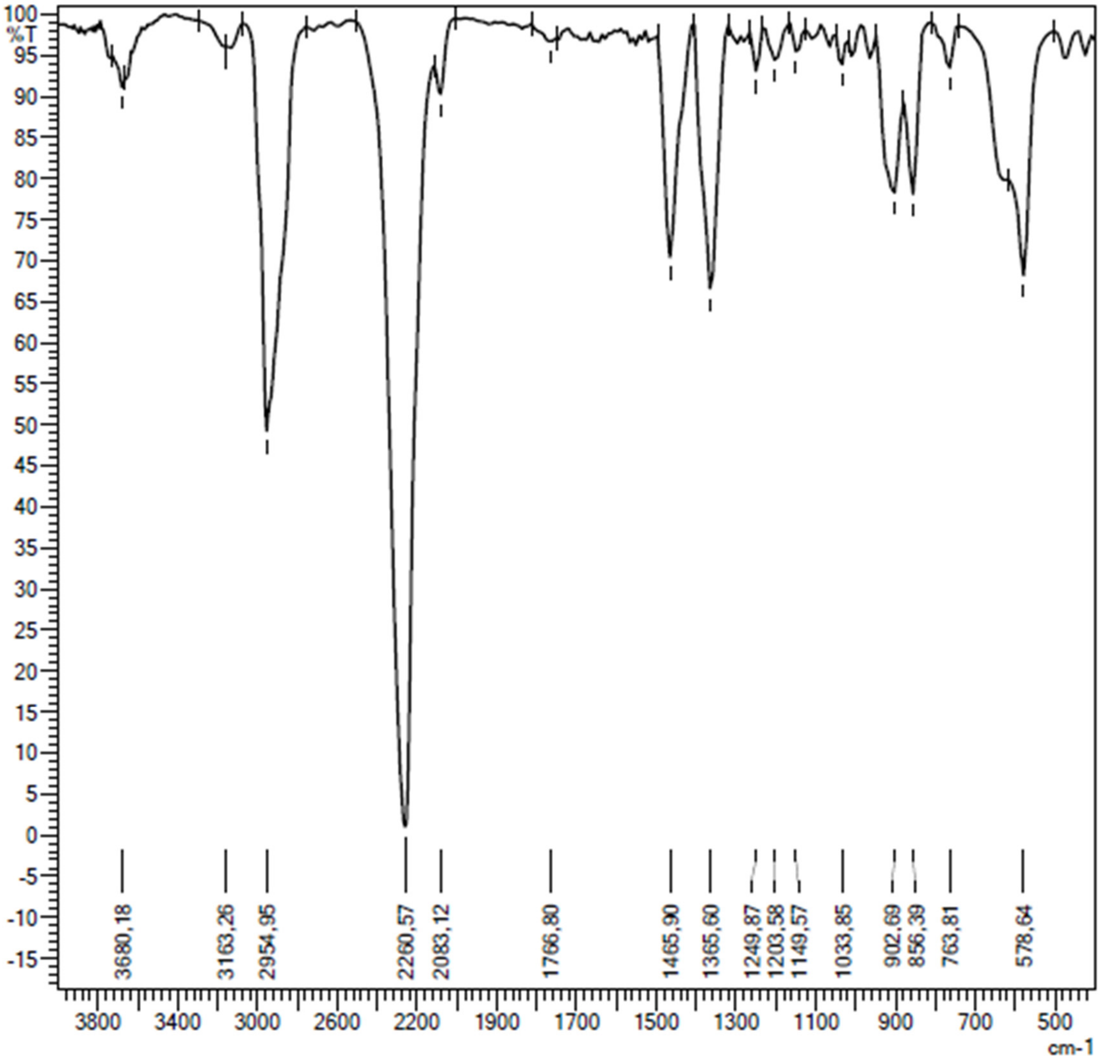

Group (stretching)	Absorption peak (cm^{-1})
C-H	2954
N=C=O	2260

FT-IR spectrum of 4,4'-methylenedicyclohexyl diisocyanate (H_{12}MDI) (4)

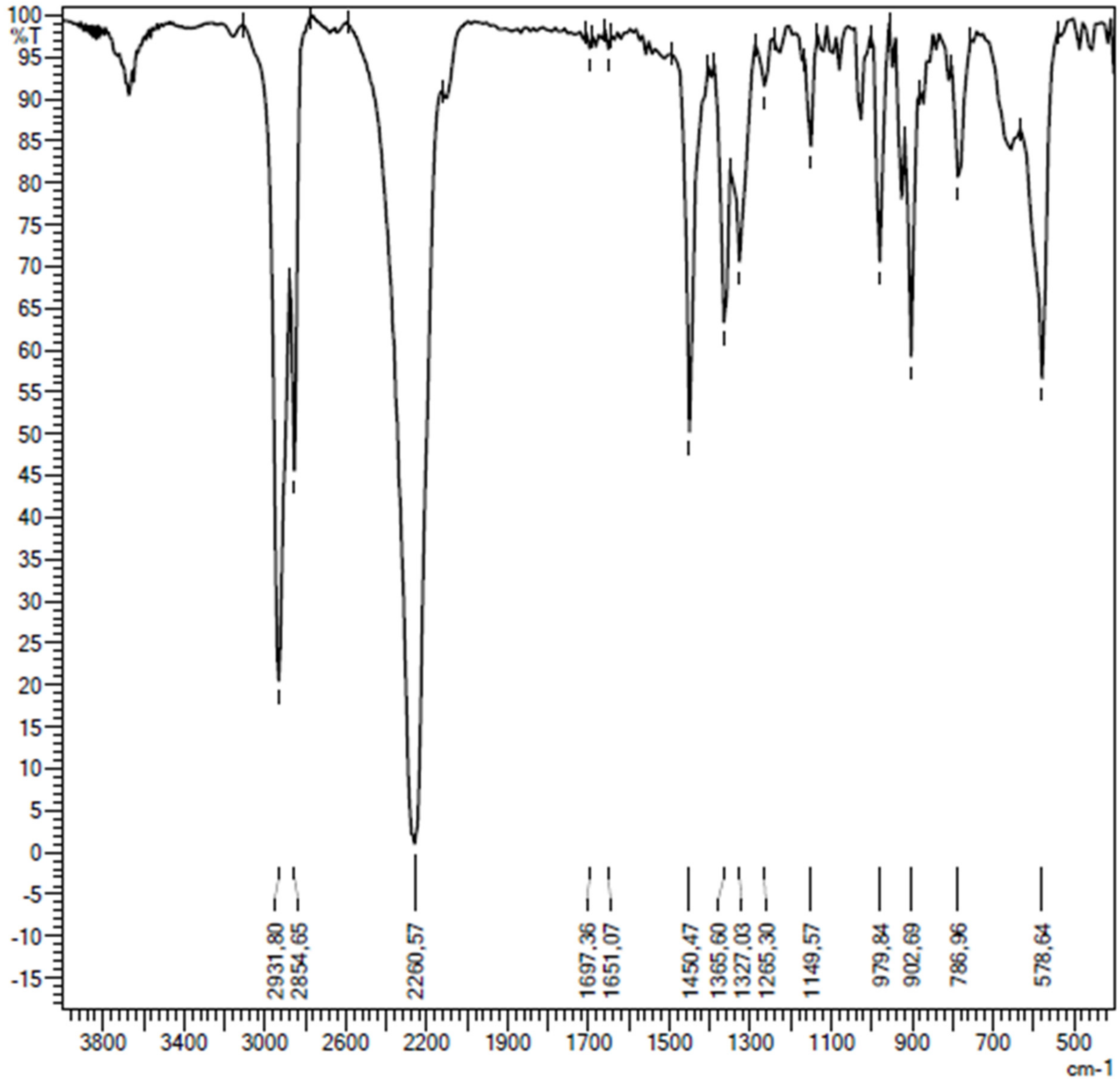

Group (stretching)	Absorption peak (cm^{-1})
C-H	2854-2931
N=C=O	2260

FT-IR spectrum of 2,2,4-trimethyl hexamethylene diisocyanate (TMDI) (5)

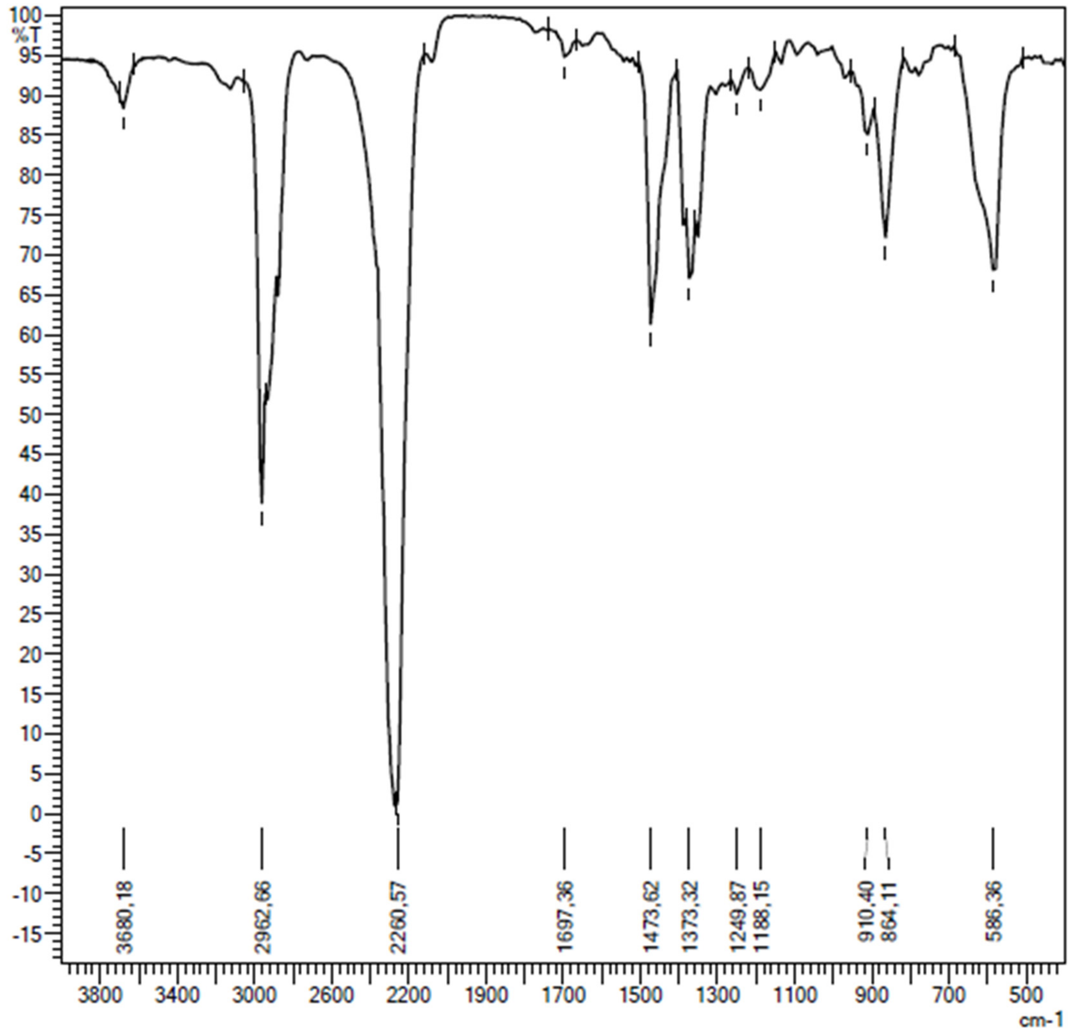

Group (stretching)	Absorption peak (cm^{-1})
C-H	2962
N=C=O	2260

FT-IR spretrum of the prepolymer (6)

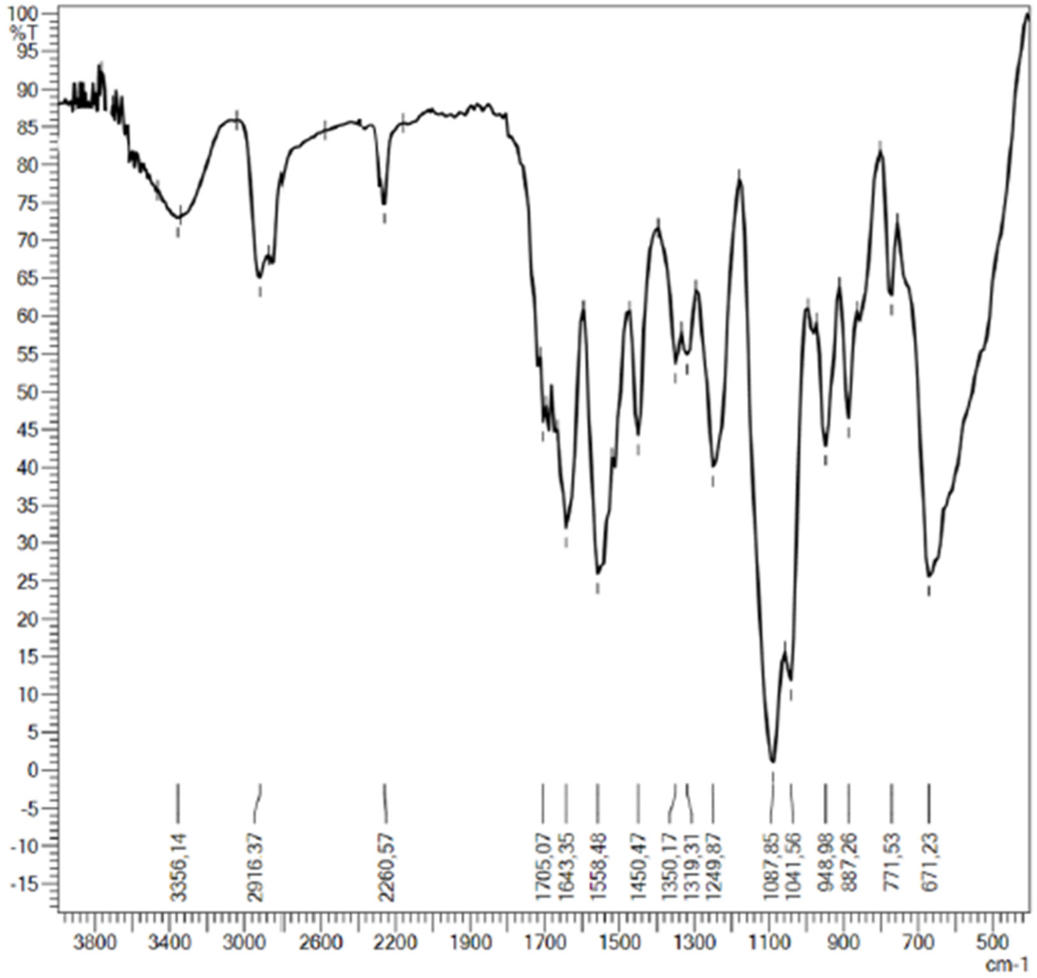

Group (stretching)	Absorption peak (cm^{-1})
N-H	3356
C-H	2916
N=C=O	2260
C=O (urethane)	1705
C=O (disubstituted urea)	1643
CO-NH	1558
C-O	1249
C-O-C	1087

FT-IR spectrum of the prepolymer (7)

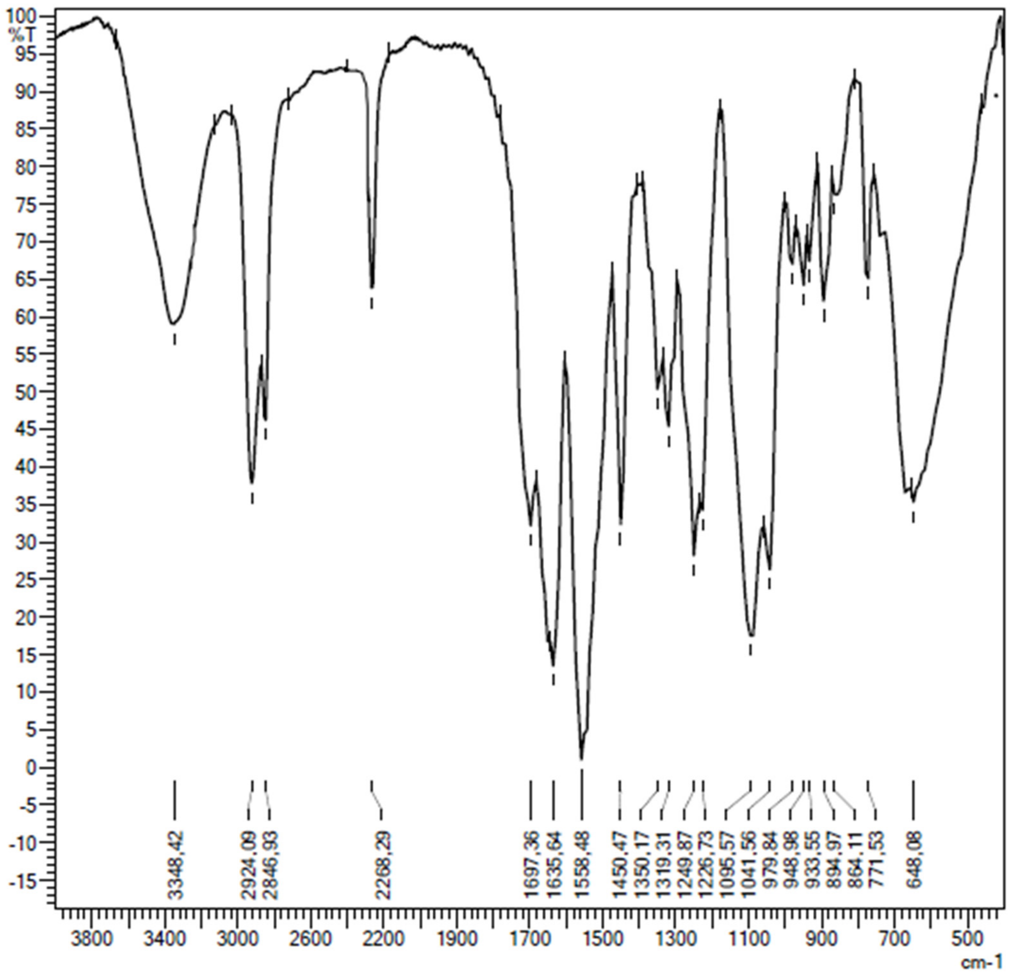

Group (stretching)	Absorption peak (cm^{-1})
N-H	3348
C-H	2864-2924
N=C=O	2268
C=O (urethane)	1697
C=O (disubstituted urea)	1635
CO-NH	1558
C-O	1249
C-O-C	1095

FT-IR spectrum of the prepolymer (8)

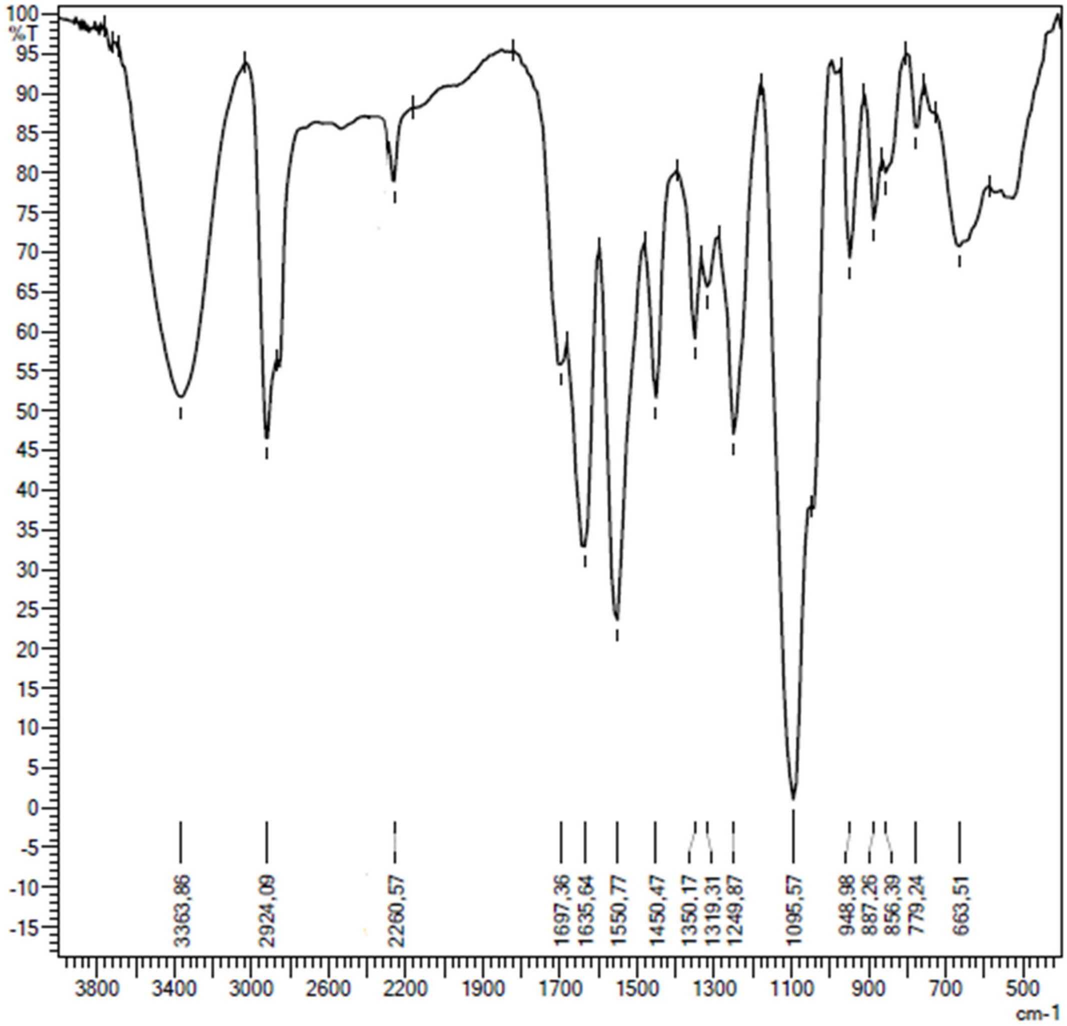

Group (stretching)	Absorption peak (cm^{-1})
N-H	3363
C-H	2924
N=C=O	2260
C=O (urethane)	1697
C=O (disubstituted urea)	1635
CO-NH	1550
C-O	1249
C-O-C	1095

Section S3. FT-IR spectra of biomass derived polyol (1)

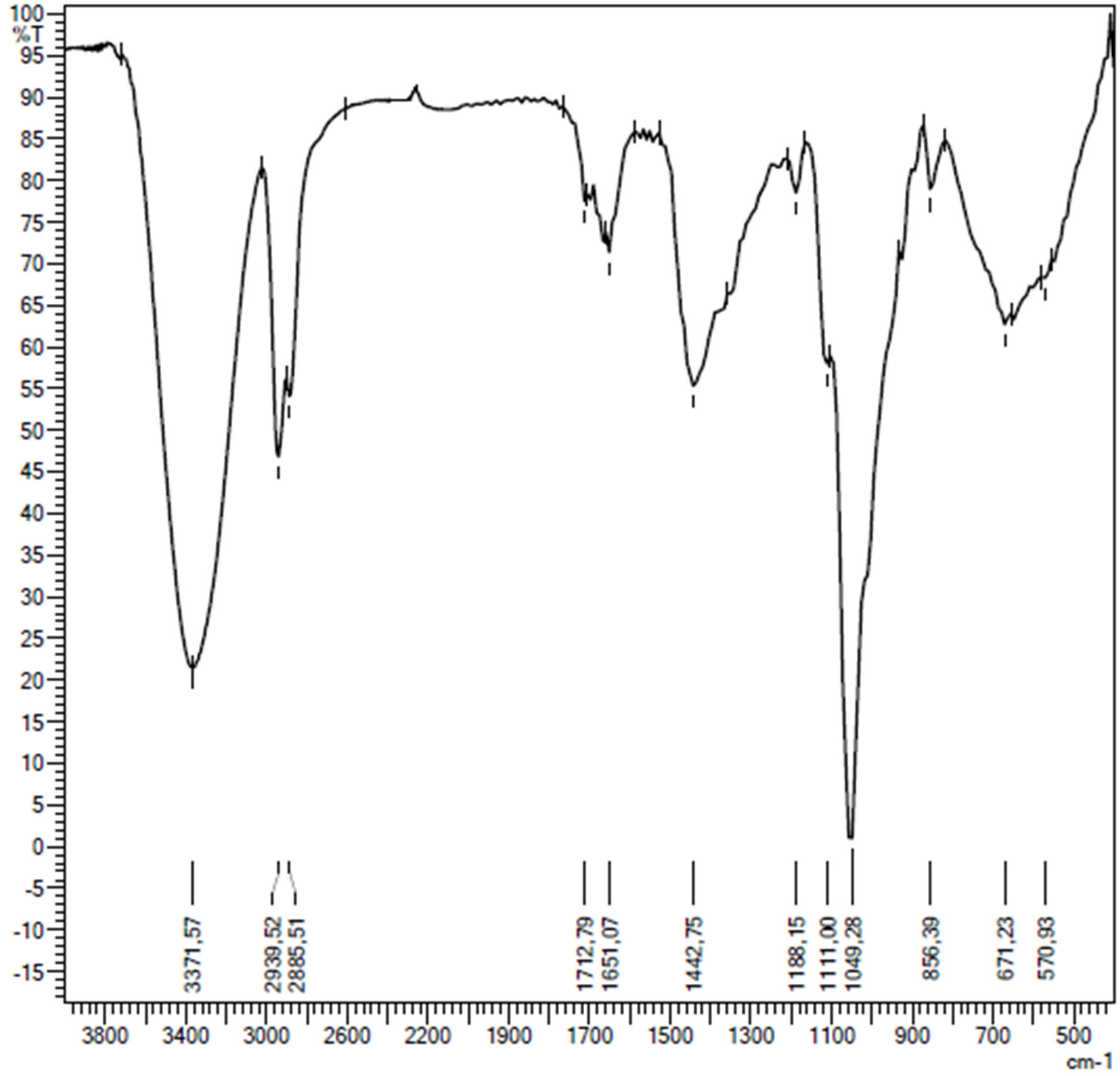

Group (stretching)	Absorption peak (cm^{-1})
O-H	3371
C-H	2885-2939
C=O	1712-1651
C-O	1188-1049

Section S4. LC-MS spectrum of polyol (1)

Full scan 70-210

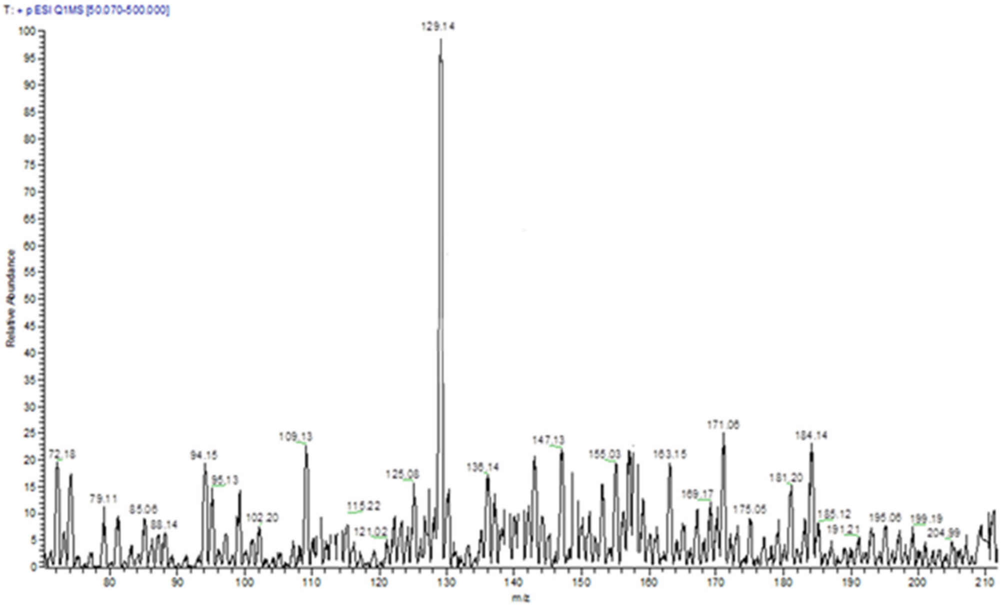

Structure	
m/z + 1	129.14

Full scan 220-250

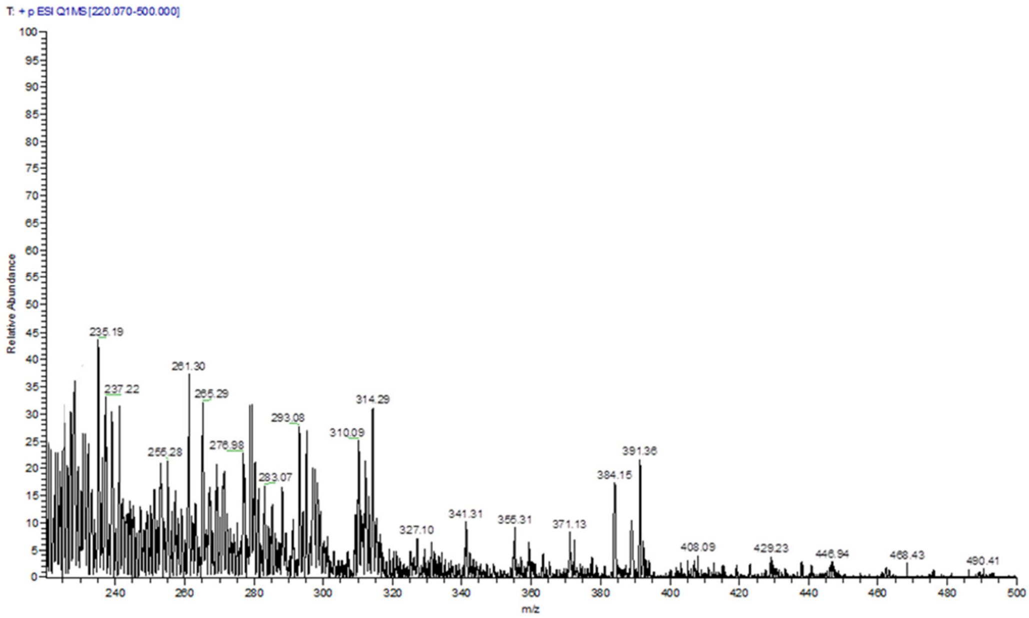

Section S5. FT-IR spectra of biomass-based polyurethanes

FT-IR spectrum of polyurethane (9)

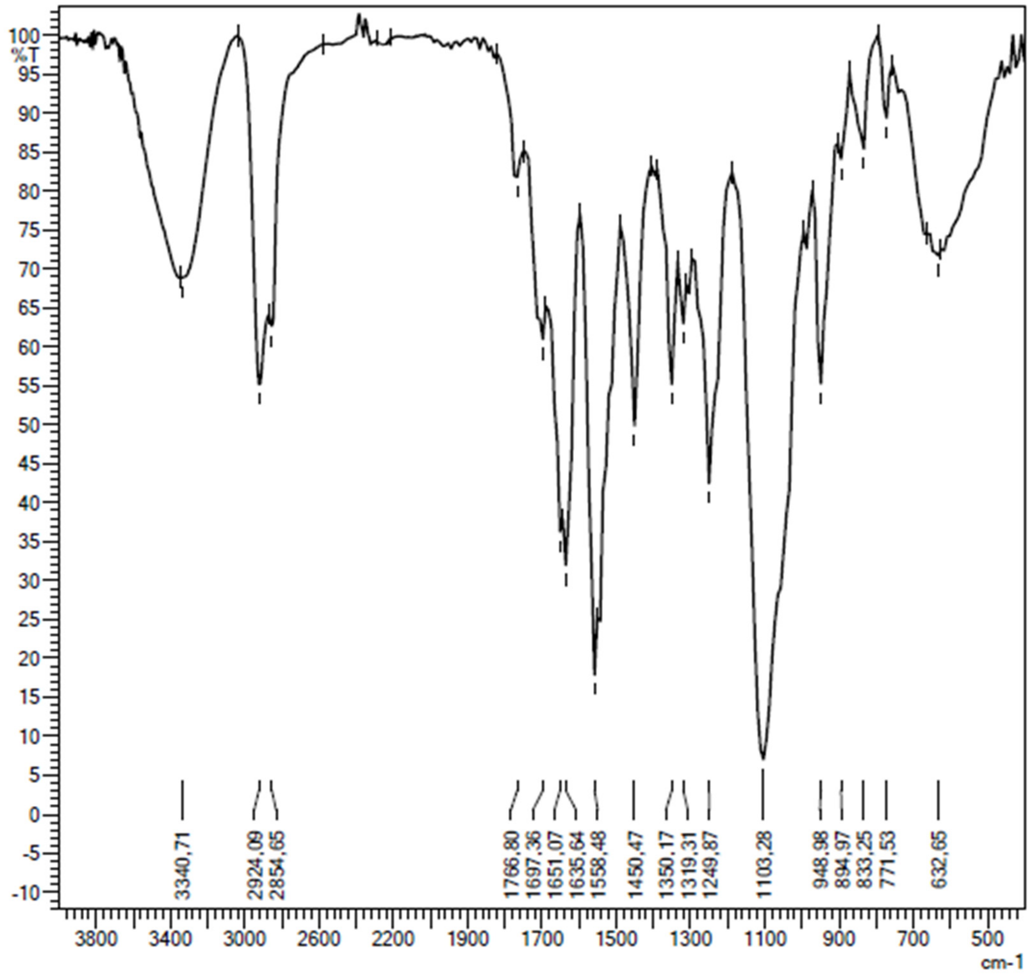

Group (stretching)	Absorption peak (cm^{-1})
N-H	3340
C-H	2854-2924
C=O	1651-1712
C-O	1188-1049

FT-IR spectrum of polyurethane (10)

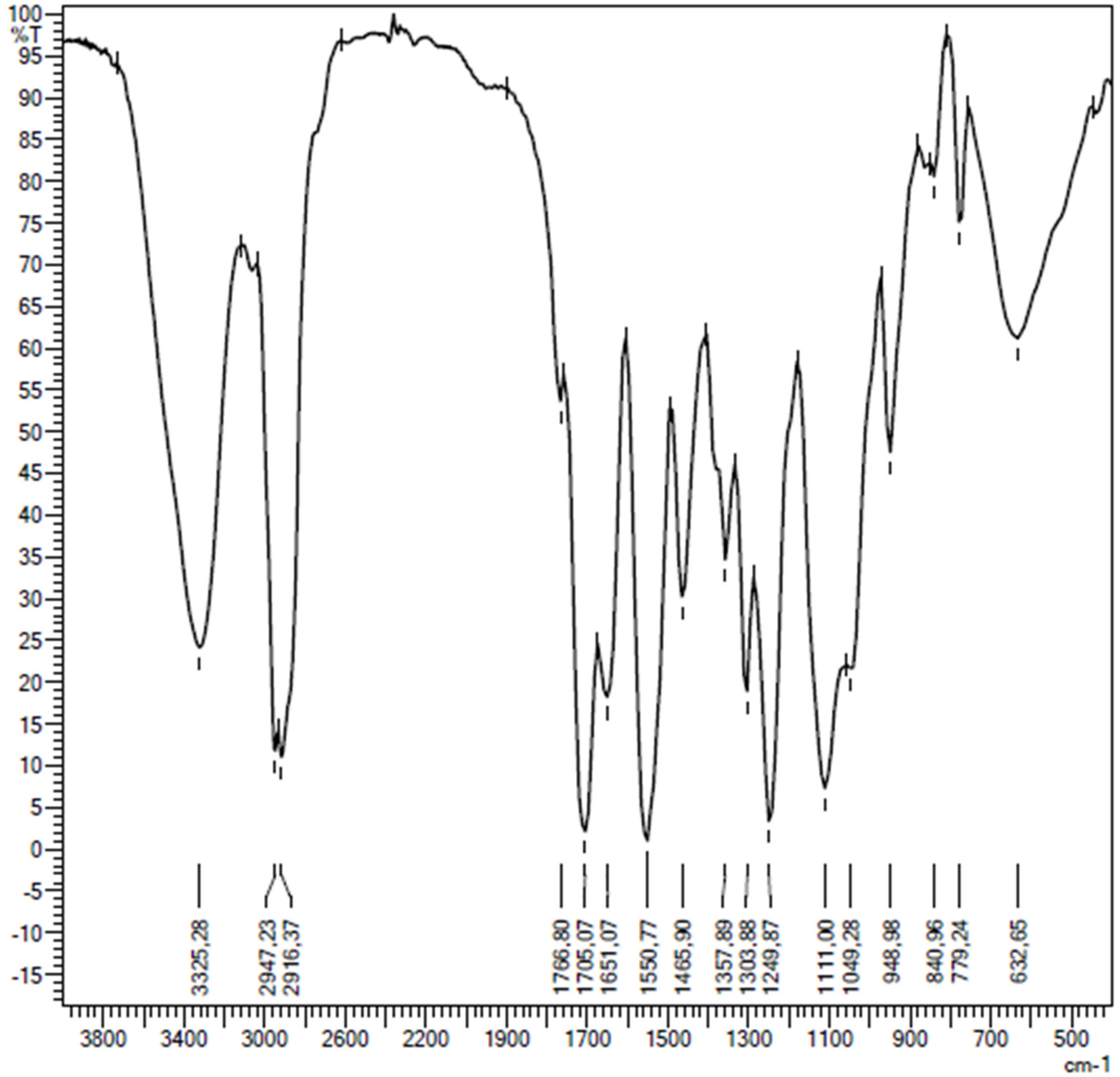

Group (stretching)	Absorption peak (cm^{-1})
N-H	3325
C-H	2947-2916
C=O	1651-1712
C-O	1188-1049

FT-IR spectrum of polyurethane (11)

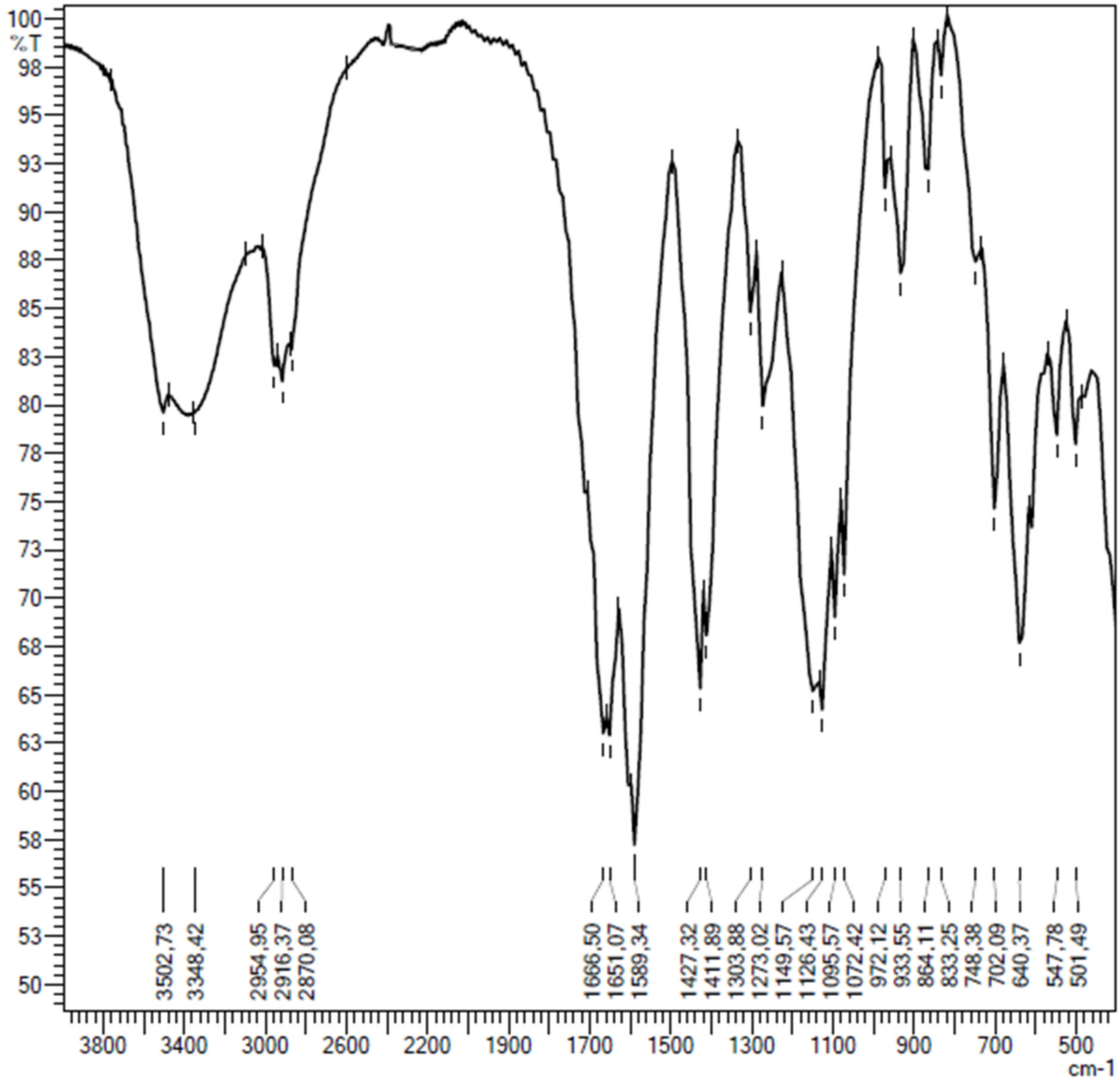

Group (stretching)	Absorption peak (cm^{-1})
N-H	3348-3502
C-H	2870-2954
C=O	1651-1712
C-O	1188-1049

FT-IR spectrum of polyurethane (12)

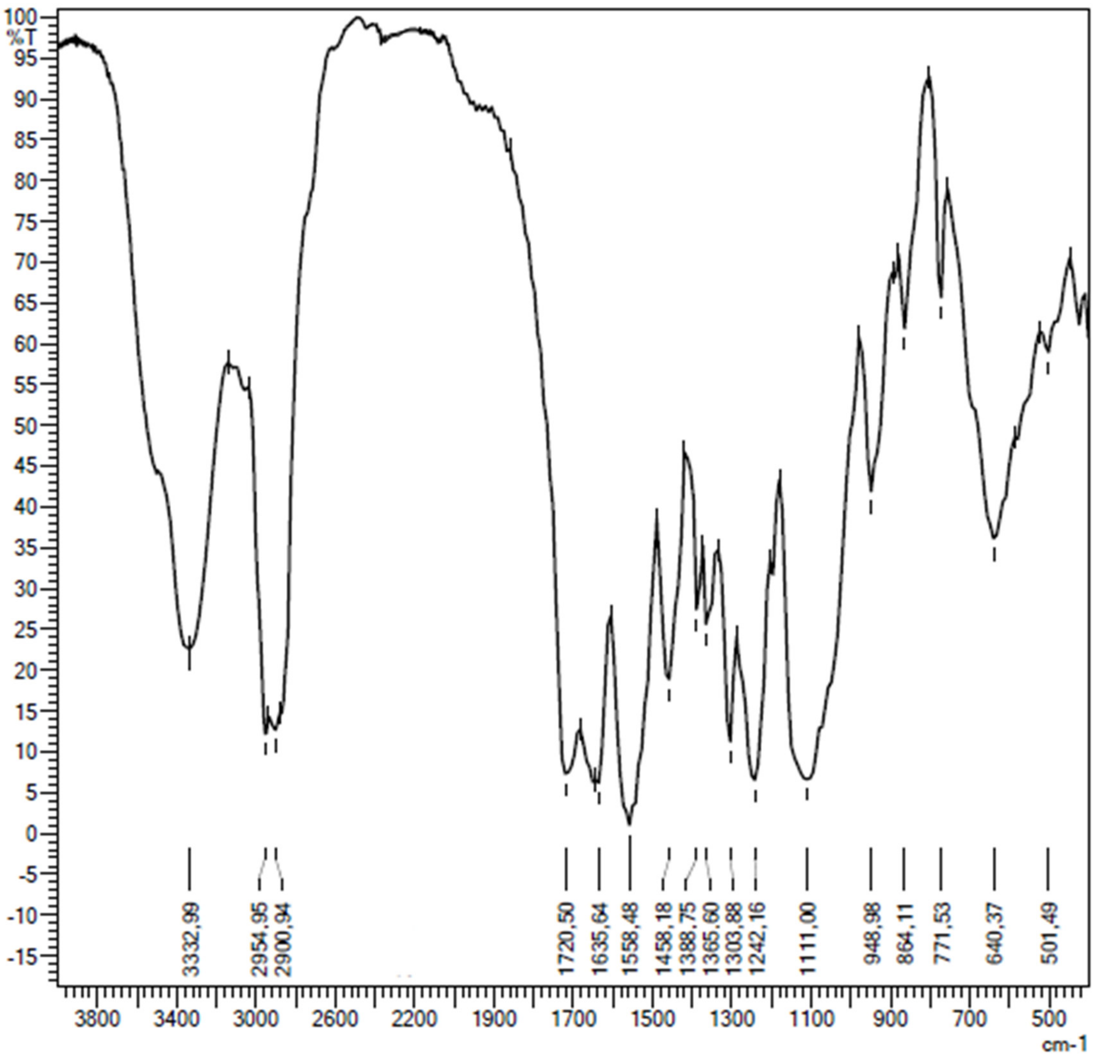

Group (stretching)	Absorption peak (cm^{-1})
N-H	3332
C-H	2900-2954
C=O	1630-1735
C-O	1188-1049

FT-IR spectrum of polyurethane (13)

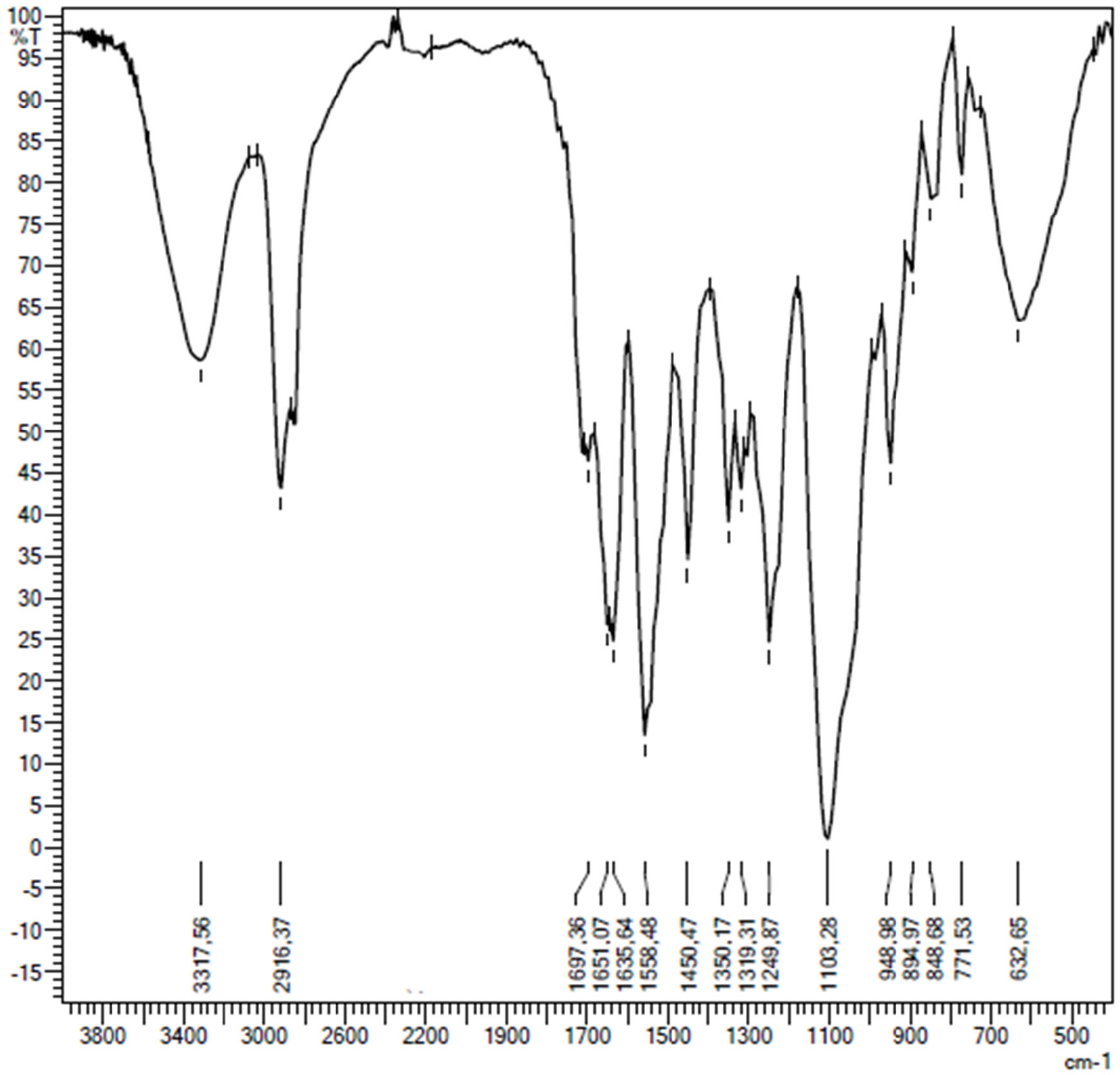

Group (stretching)	Absorption peak (cm^{-1})
N-H	3317
C-H	2916
C=O	1630-1720
C-O	1188-1049

FT-IR spectrum of polyurethane (14)

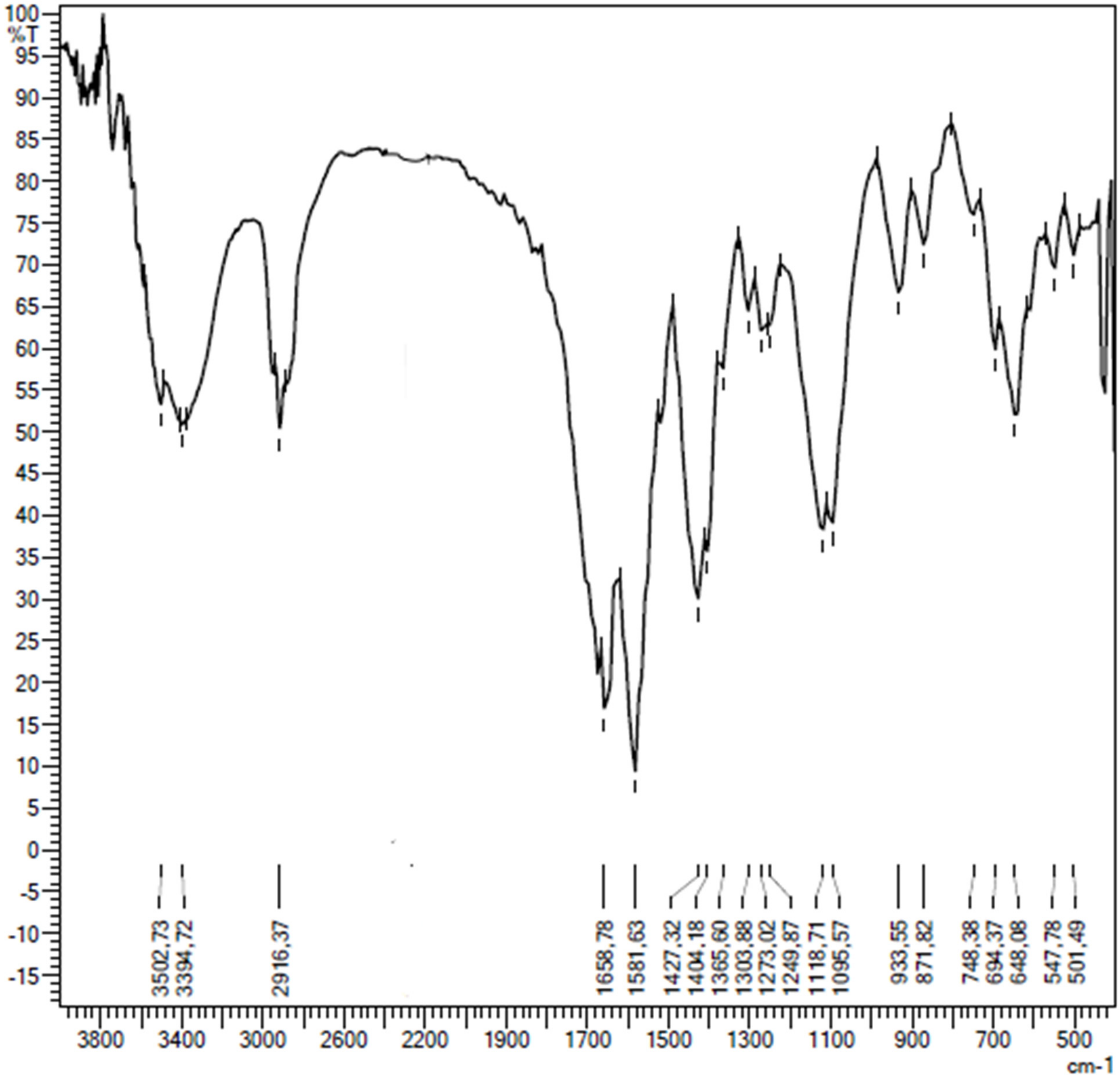

Group (stretching)	Absorption peak (cm^{-1})
N-H	3394-3502
C-H	2916
C=O	1651-1720
C-O	1188-1049

Section S6. Composite material processing equipment

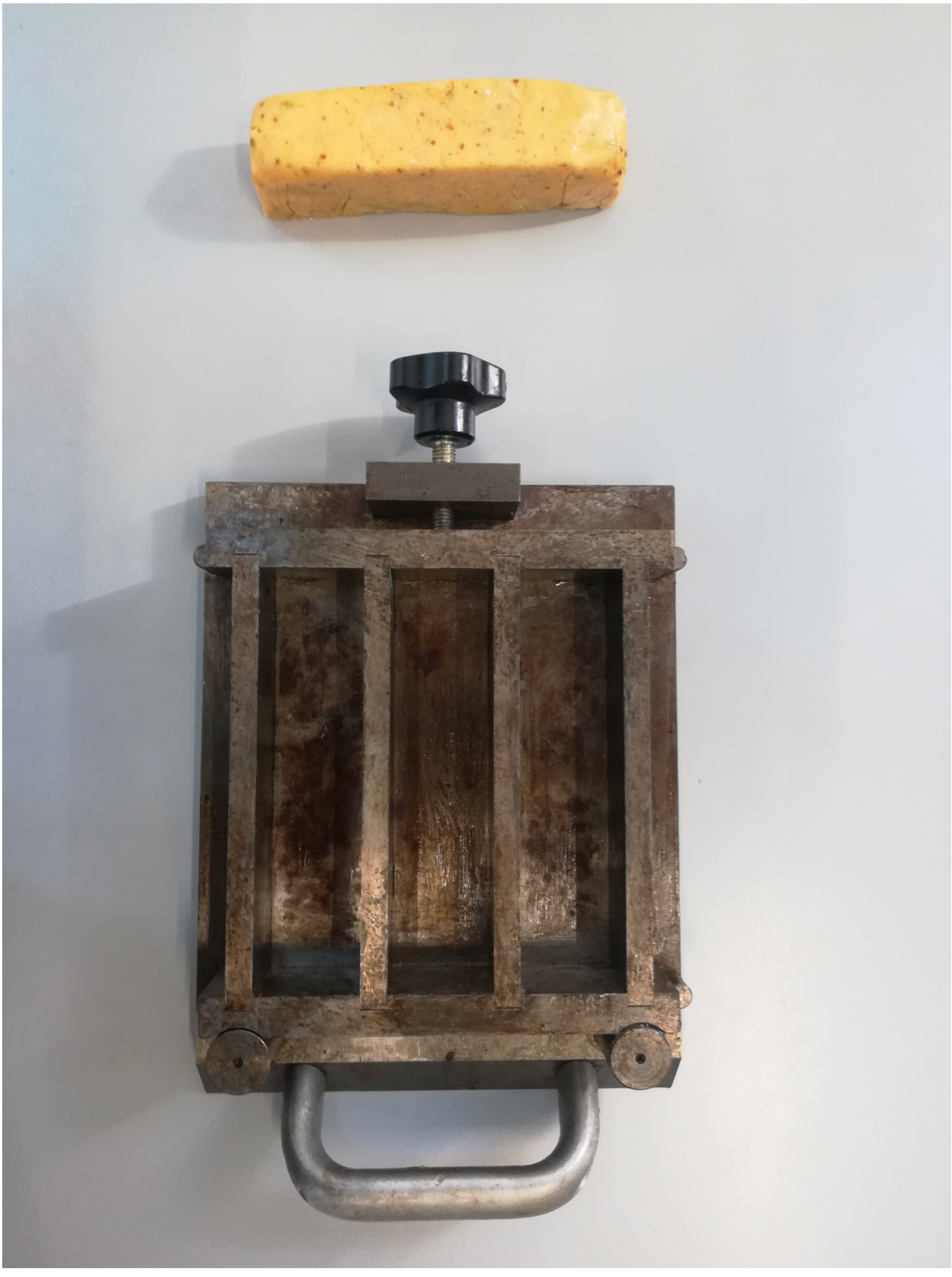

Section S7. Photographs of bio-based polyurethanes

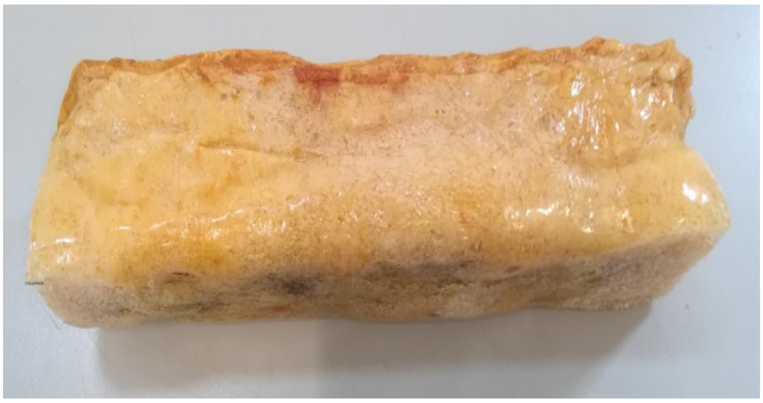

9 (rigid foam)

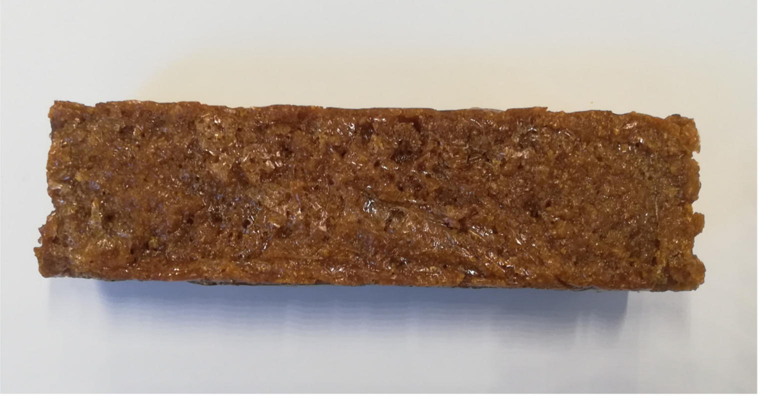

10 (rigid foam)

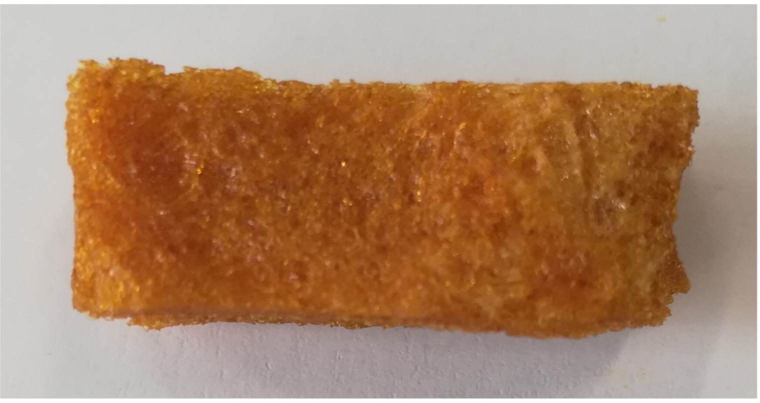

11 (flexible foam)

Section S8. Photographs of bio-based composite materials

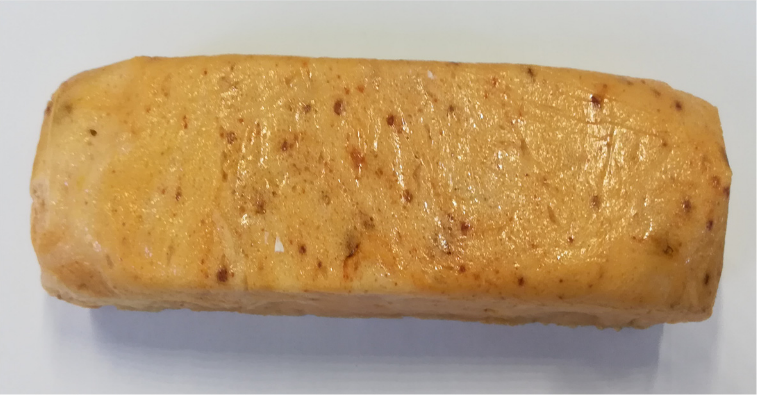

12 (rigid foam)

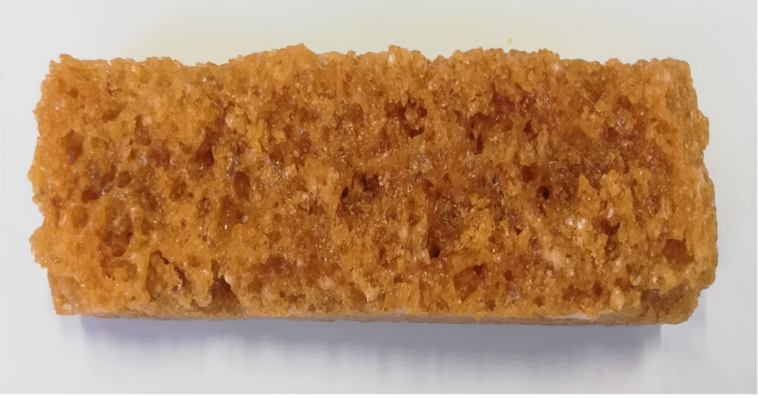

13 (rigid foam)

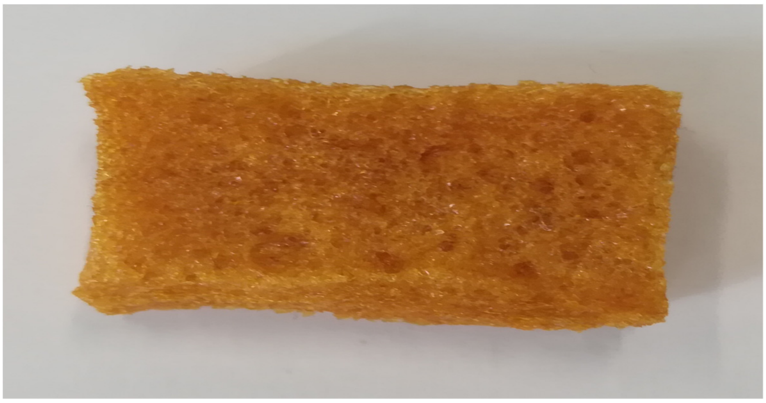

14 (flexible foam)
